# From Public Eugenics to Private Eugenics: What Does the Future Hold?

**DOI:** 10.5935/1518-0557.20220032

**Published:** 2022

**Authors:** Vera Lúcia Raposo

**Affiliations:** 1 Faculty of Law of Coimbra University, Coimbra, Portugal

**Keywords:** gene editing, offspring selection, private eugenics, public eugenics, reproductive rights, reproductive techniques

## Abstract

Traditional public eugenics, which was ordered by the State, has been replaced by a kind of private eugenics conducted by parents using reproductive techniques, genetic testing and, eventually in the future, genetic engineering. While traditional eugenics strived to improve the species, the new model aims to satisfy parents’ reproductive aspirations. The association between public and private eugenics is an ongoing issue, mostly due to its relation to nazi eugenics. This paper will state that both are eugenics; however, with different characteristics, and thus worthy of different legal and ethical assessments. The paper will contextualize private eugenics in the framework of reproductive rights (legal and ethical perspective) and in the development of genetics and reproductive techniques (scientific perspective). Finally, it will analyze some of the legal consequences of a broader acceptance of private eugenics, namely in terms of liability and tort law. Throughout the paper, the different legal solutions in place in Europe will contextualize its considerations.

## INTRODUCTION

Parental reproductive choices differ from traditional eugenics, aimed at creating better human beings, because some of the traits selected by parents for their children are not necessarily linked to the general concept of perfection. Nonetheless, since parents may believe they are improving their offspring with these choices, this paper refers to this kind of selection as private eugenics.

This paper will analyze the dichotomy public eugenics *versus* private eugenics, and from there develop the notion of private eugenics, its content, and consequences.

## EUGENICS

### Eugenics in Human History

Humans are not all born with the same genetic endowment. Some are stronger, faster or healthier, and others are more intelligent than their peers. Individuals have different abilities to adapt and survive, and only part of humanity is likely to endure. Eugenics, which aspires to create humans that are fit for survival, is a scientific doctrine advocating for more ‘able’ human beings as opposed to those who are feeble. By using eugenics, natural selection is replaced by a new kind of artificial selection determined by man.

The intent to create superior humans is neither new, nor a product of modernity. Since the beginning of time, the human species has shared a dream of perfection (Suter, 2007). Historians have told us that in the ancient Greek city of Sparta, the weaker babies were killed at birth. Even the great philosopher Plato argued that every society should be ruled by a superior class that reproduces and grows, disseminating its better-quality genes throughout the community. In the nineteenth century, Charles Darwin advanced a theory of human advancement based on the evolution of the human species in his book *The Origin of Species*. Thought it was not a theory about human enhancement, it served as a basis for modern human enhancement theories. His theory was followed by Francis Galton, who named this idea eugenics, from the Greek word for ego, eugenés (êu-good, génos-race). The term appeared in written form for the first time in Galton’s 1883 work, *Inquiry into the Human Faculty and its Development,* transforming him into the father of modern eugenics (Campiglio, 2003; Cavaliere, 2018 ).

As of the beginning of last century, several countries began to include eugenics guidelines into their policies, and various forms of legislation incorporated these concerns. However, the most dramatic cases of eugenics were found in Nazi Germany. Their policies of racial hygiene-imposed sterilization on mentally ill people, condemned sexual union between members of the Aryan ‘race’ and other ‘races’ reputed to be inferior; and ultimately, resulted in the practice of euthanasia on the grounds that some lives did not deserve to be lived. These events created a strong movement against eugenics, in which its contrariety with the principle of human dignity was invoked, but there is an on-going discussion on the relation between human dignity and eugenics (Beyleveld & Brownsword, 2001; Raposo, 2019).

However, the eugenics movement, is much more complex than the simplistic racist experiment carried out by the Nazis, and it can assume various forms, not all of which deserve the same legal or ethical assessment.

Positive eugenics refers to the amelioration of the species through the reproduction of its ‘ameliorated’ members, either by stimulating sexual intercourse between them or making use of technological methods to facilitate the propagation of their genes (such as the selection of gamete donors according to their genetic potentialities). Another scientific technique, genetic engineering, maximizes one’s genetic prospects, (i.e., the addition, substitution or deletion of genes to create a more enhanced human being) and it can also be used for positive eugenics. In short, positive eugenics aims at improving existing (e.g., gene editing in in vitro embryos) or prospective human life (e.g., reproduction using selected gametes), and it does not involve the elimination of human life (Anomaly, 2020).

Conversely, in negative eugenics the weaker elements are restrained from procreating (for instance, through coerced sterilization or abortion) to eliminate defective genes and thus “clean” society and, in the most drastic scenarios, negative eugenics involves the killing of the weakest elements. Like positive eugenics, scientific methods have enabled a new form of negative eugenics, by diagnosing *in vitro* embryos and suppressing those that are deemed less fit.

### Private Eugenics versus Public Eugenics

In the last decade a different type of eugenics came to the spotlight, it’s been called new eugenics (Daar, 2017), or neoeugenics (Suter, 2007), sometimes referred to as private eugenics (Gupta, 2007) or domestic eugenics (Dantas, 2008). Some fancier expressions have been used to distance new eugenics from old eugenics, such as ‘procreative beneficence’, an expression created by Julian Savulescu to describe the parental obligation to improve the genetic qualities of offspring (Savulescu, 2001; Savulescu & Kahane, 2009).

While in the past eugenics was essentially a public concern, even one imposed by the State, this new modality is a private concern: parental choice over their offspring’s characteristics (Galton & Galton, 1998; Gumer, 2019; Lou, 2015; Kevles, 2016; Mohapatra, 2016; De Paor & Blanck, 2016).

Traditional public eugenics had two goals: on the one hand, and primarily, to eliminate the weakest, ultimately by killing them (negative eugenics); on the other hand, it also sought to cultivate strength (positive eugenics). This was closely aligned with the purity of race argument, and perhaps explained its racist connotations (Mittra, 2007).

In contrast, private eugenics is not aimed at the death of anyone (rectius: it does reject pathological material and strives to prevent the birth of undesired children; however, it does not destroy life that already exists). Some have accused private eugenics of being a tool of discrimination (Bachrach, 2004), which might end up being truth, if parental selection deals with a specific gender, race or ethnicity (Russell, 2021)_._ The words of the Supreme Court Justice Clarence Thomas on the matters of the Indiana law on abortion are clear in this regard:

Each of the immutable characteristics protected by this law can be known relatively early in a pregnancy, and the law prevents them from becoming the sole criterion for deciding whether the child will live or die (…) Put differently, this law and other laws like it promote a State’s compelling interest in preventing abortion from becoming a tool of modern-day eugenics.

However, discrimination is not a necessary consequence of eugenics (Raposo, 2022). Private eugenics is also used to promote the birth of healthy children - preimplantation genetic diagnosis (PGD) to select healthy embryos, the use of non-infected gametes - that can expect a pain-free life, instead of the limited existence that they would expect otherwise.

Public eugenics has been accused of violating the fundamental right to reproduce and create a family, because its measures have been imposed by the State (Suter, 2007). Some have supported its imposition based on a higher value, namely a public interest in reducing the number of citizens dependent on the State and saving humanity from extinction (Smith, 2000). In contrast, private eugenics has grown out of individual freedom, based on parental choice, sometimes in opposition to the State’s intention to limit it. The discussion about the limits and the grounds for the State to limit parental choices in this regard is current among scholars (Gyngell & Douglas, 2015). While some conclude that ‘it is legitimate for the state to intervene in the genetic supermarket to prevent collective action problems’ (Gyngell & Douglas, 2015), others advocate in favor of broader reproductive freedom (Harris, 1998; Robertson, 2008).

Historically, public eugenics could prevent undesirable conceptions by restricting marriage between people with unwanted genes. Science eventually evolved to include genetic monitoring, followed by counselling and even compulsory public decisions over reproduction imposed by the State, which became highly contested. The most drastic solution eliminated people qualified as unfit. The assessment of who should be deemed unfit was made by the State based on predominant social conceptions and varied according to time and place, but such assessment usually referred to medical conditions (physical or mental) considered undesirable.

Alternatively, private eugenics has been grounded in reproductive techniques and associated scientific procedures combined with the expansion and profitability of the ‘baby business’ (Schurr, 2018). A commonly used mechanism is the selection of genetic material with qualities appreciated by parents (Buchanan *et al.,* 2000). This is not new. Superior genetic material has also been used in traditional eugenics. However, in the past it included sexual matching between selected couples, whereas today gametes are usually selected from anonymous donors (Committee on Social Affairs & Health and Sustainable Development, 2019). In addition, PGD plays an important role in embryonic selection and recent developments in gene therapy (Grant, 2016; Rodriguez, 2016; Niu *et al*., 2014) may completely change a parent’s ability to shape his or her offspring (Raposo, 2021a).

Another difference between traditional and new eugenics is that parental choice is portrayed as essentially neutral in terms of its moral value, unlike what occurred under Nazism. However, this may not be entirely correct. Parents express certain preferences that obviously have moral value, as pointed out by Dov Fox (2016). For Fox (2016) the reason is that

It is not just that parents’ preferences for future children without certain traits implicate judgments about people who exist with those traits today. The very commitment to offspring selection is itself not value-neutral: It privileges the value of parental control over the value of parental acceptance

The aim is not to disparage people living with undesired characteristics, but to support parental control over the reproductive process. The supporters of private eugenics contend that parents should be able to choose the kind of child they want, just as they can decide what kind of education their child should receive. In other words, they understand reproduction within the framework of parental autonomy (Raposo, 2021b). Going a step further, they assert that the decision over what kind of child to have is just as relevant as the decision over whether to have a child at all (Botkin, 1995), and thus falls within the scope of reproductive rights protection (Robertson, 1994; 1996).

### The vilification of eugenics

In spite of their differences, both public and private practices are eugenics (De Paor & Blanck, 2016). Some might refrain from qualifying parental reproductive choices as private eugenics because of the horrors usually associated to this word. Eugenics has been vilified. But even though the past showed us so many undignified models of eugenics (the Nazi doctrine is paramount), this does not imply that all eugenics are necessarily bad (Caplan *et al*., 1999; Anomaly, 2018).

The kind of eugenics we use today, associated to reproductive techniques and related scientific procedures, aims to promote the wellbeing of specific children, and ultimately of current and future generations (Raposo, 2019; Root, 2000). It is a mechanism for disease prevention and to spare people from future pain (Raposo, 2017a; 2017b). By using different scientific and medical procedures, parents can promote the birth of a healthy child, even if with that choice they prevent the birth of an unhealthy child. The being that was not born does not suffer any injury. There is no violation of its right to be born, because only a legal person can claim such a right, and (at least in Europe) the unborn is not a legal person (Engelhardt, 1976; Mori, 1996), as confirmed by several legal standards (see Article 66 of the Portuguese Civil Code and Articles 29 and 30 of the Spanish Civil Code). So, by preventing the birth of a non-healthy child, and instead promoting the birth of a healthy child, parents are actually proving a better chance of life to the person that is effectively born (Savulescu, 2001).

### Modalities of Private Eugenics

Parental choice over their offspring’s characteristics can follow three different paths, each of which deserves its own legal and ethical assessment.

First, parents can select health related characteristics, that is, the ones that promote the birth of human beings free of pathological traits. The prevention of pathological features (although the notion of disease is not completely clear, as we shall see) is one of the main reasons grounding parental decisions to terminate pregnancy, or to use reproductive techniques to prevent the birth of a child with certain characteristics. However, it is not the only one. In several European jurisdictions the termination of pregnancy is allowed when the mother’s life or health is at risk, when a sexual crime has been committed against the mother, or when the woman’s reproductive freedom forms the basis of the decision to terminate the pregnancy (Italy, Portugal, Spain) (Lavelanet *et al*., 2018). Likewise, in many European jurisdictions reproductive techniques can be used for different reasons, not only to enable reproduction of infertile people, but also to assist single people (Volgsten & Schmidt, 2021) or gay couples (Tam, 2021), or to prevent the transmission of a hereditary or infectious disease (Richardson *et al*., 2015). Around the world we can find different solutions, some more liberal, others more restrictive. In any case, the prevention of pathological features tends to be a common justification either for abortion or the use of reproductive techniques.

This type of selection has been corroborated by several court decisions in Europe. Abortion based on an unborn’s health condition has been widely accepted, even in more restrictive jurisdictions in Europe, such as Poland and Ireland. Malta, Andorra and San Marino are one of the few that still imposed a strict ban (Center for Reproductive Rights 2021). The use of PGD to select healthy, *in vitro* embryos is also generally allowed. Even though gender selection is banned in Europe, national regulations permit the selection of a specific gender to avoid a disease associated with the opposite gender (Duguet & Boyer-Beviere, 2017). The same solution results from Article 14 of the Convention for the Protection of Human Rights and Dignity of the Human Being with regards to the Application of Biology and Medicine: Convention on Human Rights and Biomedicine:

The use of techniques of medically assisted procreation shall not be allowed for the purpose of choosing a future child’s sex, except where serious hereditary sex-related disease is to be avoided.

One of the main reasons why the use of donated gametes is legally allowed is to avoid the transmission of a medical condition. This is not the only reason; however, because under some laws in place in Europe, donated gametes can also be used to enable the reproduction of single people or gay couples. Conversely, gamete selection in Europe cannot be aimed at achieving non-healthy-related outcomes for the future child. In the UK the use of mitochondrial material from a donor is now expressly allowed for the same reason.

Furthermore, although genetic engineering has not become a commonly accepted practice, its connection to health may generate legal support. In Europe, there is disagreement over this practice. Several national and international norms ban some types of genetic manipulation. It is not clear if genetic manipulation should or should not be accepted, but the discussion is much more vivid regarding non-health-related manipulations. In contrast, health-related interventions have gained greater support, especially in light of Article 13 of the Convention on Human Rights and Biomedicine:

An intervention seeking to modify the human genome may only be undertaken for preventive, diagnostic or therapeutic purposes, and only if its aim is not to introduce any modification in the genome of any descendants.

In sum, among all of the hypothetical reproductive choices parents can make, those related to health are the most commonly accepted under European national and international regulations.

Second, parents can select features that are objectively harmful to an individual (painful diseases or other traits, such as deafness or dwarfism, whose negative value is far more controversial), or refuse to use methods that would prevent the transmission of pathologies to offspring (for instance, by declining to use donated gametes or conducting PGD; both methods are aimed to avoid the birth of people with those features). Scholars are divided regarding the admissibility of parental reproductive and genetic decisions that cause objective harm to the child; however, laws and judicial rulings tend to ban such decisions.

Third and finally, parents can select features that are neither clearly health-related nor clearly harmful to the future person, such as eye color, hair color or gender (when not associated with a medical condition, otherwise this is viewed as a health-related selection). Some of these traits are objectively valuable (for instance, higher intelligence) and are a form of human enhancement (Raposo, 2022). Others are neutral (even futile, in the sense that the characteristics selected are considered irrelevant for the child’s future) (Naik, 2009), under the reasonable person standard; however, they seem to be important to some parents (Raposo, 2021a) (take the case of gender: it has been so important in certain cultures that it has led to selective abortion and even to the infanticide of female babies).

### Private Eugenics and the Myth of Perfection

In our regular lives we try to perfect ourselves. We go to libraries and universities to develop our intellectual capacities. We exercise to gain good physical condition. We wear sophisticated clothing and makeup to attract sexual partners we consider to be good matches for procreation, expecting to have children that will inherit a genetic patrimony we consider to be of ‘high quality’ (Russell, 2021). In short, we tend to try to maximize the capacities with which we were born (Healy, 2020). The difference is that today we can accomplish this by resorting to science and genetics (Rodrigues *et al*., 2020), and these new methods, aimed at achieving old purposes, guarantee a level of success unknown until now.

However, the paradox is that what is considered perfection does not last long. Whenever a certain characteristic, previously praised because of its rarity, becomes a common feature its value decreases. Singer (2003) uses the example of height and talks about an escalating height race in which the selection of increasingly tall children might change what is considered normal in terms of height. So, the pursuit of perfection is illusory because when reached, it stops being perfect.

It can be argued - as Sandel (2004) does - that parents should not rely on the myth of perfection as their reproductive goal. According to this reasoning, the motive to have children is the children themselves and the desire to provide the best for them, not to fulfil a parent’s personal motivation. We can even discuss the existence of a parental duty to accept any feature the child might have and provide him/her with unconditional love (Malek, 2013). However, even accepting these premises - debatable premises, but not the object of the present analysis - this does not mean that, taking into consideration the good of the future child, parents cannot also envisage fulfilling their own desires (Benatar & Wasserman, 2015), such as creating a child that reaches their idea of perfection. There is really no way to avoid this, and it would be naïve to assume that (all) parental decisions, prior to and after birth, are exclusively geared towards the child’s best interests. As Malek (2013) concludes, parents can fully accept their children and love them, but still try to select a child with certain features they highly praise because, in the end, people have children to reach their own selfish purposes (Raposo, 2014).

### Private Eugenics and Reproductive Rights

This paper does not address the existence of reproductive rights. It assumes that these rights exist, given that they are commonly recognized in jurisdictions around the world, including European. Instead, the focus of this paper is on the content of reproductive rights (Andrews & Elster, 2000), i.e., whether they include selecting offspring’s characteristics unrelated to health and not immediately detrimental to the child’s well-being.

Reproductive rights confer parents the freedom to choose whether to have children and, if so, how many. What is currently under discussion is whether this should include the freedom to select what kind of children to have. (Raposo, 2021a; 2021b).

In invoking parental freedom to make decisions on behalf of their living progeny, in specific cases, parents have been allowed to decisively influence their child’s life; for instance, by withholding consent to surgically correct a heart defect; refusing to consent to chemotherapy; denying permission for a child to be given psychotropic drugs, even though the parents no longer have custody; or donating a child’s kidney to a sibling (Mehlman, 2009). Along this line of reasoning, Mauron (1999) found that genetic interventions aimed at satisfying parents’ reproductive rights should be accepted (a kind of private eugenics), but that genetic interventions oriented to community or State interests (traditional or public eugenics) should not, independent of germinal or somatic therapy. Similarly, Ossareh (2017) found the criteria on which offspring traces can be accepted in their connection to reproductive rights: to the extent the selection of a child’s specific characteristics influences the parents’ decision to procreate. This selection falls under the scope of reproductive rights protection and so it must be respected. This thesis must be understood within the more general theory, stating that the content of reproductive rights includes the right to select offspring’s characteristics. One of the main advocates of this thesis was John Robertson (1994; 1996). In his very liberal understanding of reproductive rights the author goes even further, arguing that this concept includes access to any information relevant to deciding whether to procreate or not, as long as it is decisive enough to make parents decide whether to abort or implant the *in vitro* embryo (Robertson, 2003a). This thesis has also been sustained, even if in slightly different terms, by other authors, such as Nozick (1974), Harris (1998), Agar (2004), and more recently Ossareh (2017). In support of this position, it has been argued that such characteristics may be crucial to the reproductive decision, and that without the guarantee that a child will have the desired features, a choice may be made against having a child.

This position can be contested by a different understanding of reproductive rights, according to which the only right is to have a child, any child (eventually we can say any healthy child); but not to have a particular child (Vacco, 2005). Not even the right to make child rearing decisions, fully recognized by the US Supreme Court, has included a concomitant right to make decisions regarding a child’s characteristics (Andrews & Elster, 2000). According to this perspective, the ban on the selection of an offspring’s features does not violate reproductive rights (Coleman, 2002).

The main developments in the reproductive rights doctrine have come from the United States. Examining the Supreme Court case law, I have not found specific decisions upholding the right to use reproductive techniques or genetic engineering, even though there have been some references to a parent’s right to shape a living child, especially with regards to its care and upbringing. As Coan (2011) points out, in light of the Supreme Court’s silence (a reflection of the US Constitution’s silence) two main positions have emerged among US scholars: the libertarians favor broad reproductive liberty, including the possibility of shaping offspring’s genes (Agar, 2004; Nozick, 1974; Robertson, 1994; 1996; 2003a; 2003b; 2008) and the communitarians, restricting this possibility based on concern over the potential harm to the community of extensive procreative liberty (Kass, 2000; 2002; Sandel, 2004). However, there has been no consensus regarding the equivalence between this and the freedom to decide on an unborn’s characteristics (Gyngell & Douglas, 2015).

In Europe, in contrast, the figure of reproductive rights never received wide acclaim by Courts or scholars. This concept is mostly used to include practices such as abortion, voluntary sterilization and access to contraceptives (Policy Department for Citizens’ Rights and Constitutional Affairs, 2018), all of them dimensions of the right not to reproduce (Cohen, 2010). *A maiori, ad minus* the possibility for parents to select the feature of offspring is not discussed in the framework of reproductive rights. In light of the regulations in place in Europe, the scope of parent’s reproductive choices is very narrow and basically only covers health-related aspects (Raposo, 2017b).

### Private Eugenics, Reproductive Techniques and Associated Procedures

In a sense, parents have always practiced a kind of eugenics, starting with the choice of one procreative partner over another, and selecting a partner whose characteristics are desired for the child. The difference is that today the intervention of medicine, technology and genetics have made the selection more predictable and less dependent on uncertain genetic variations.

Reproductive techniques will not lead us back to ancient eugenics (Hoffman, 2017), but they are especially suited to private eugenics (Daar, 2017). For instance, these techniques allow the use of previously selected gametes to conceive a child with certain features. Gamete selection goes hand in hand with storing the gametes of particularly gifted donors in gamete banks, to be used to create extraordinary human beings. What appears to be scientific fiction is actually quite real. In the United States there have been reports of a so-called ‘genius sperm bank’, uniting individuals with particularly high *intelligence*, mostly Nobel laureates (see the Repository for Germinal Choice in California).

There is also an app in ITunes, called *London Sperm Bank Donors,* which allows for the selection of donors based on traits like ethnicity, occupation, personality type and eye color. In another version of gamete selection, several businesses have been created to promote egg purchases from particularly beautiful women (Martin, 2018). Another reproductive technique is PGD, enabling embryos with the desired features to be transferred to a mother’s uterus. Finally, a more radical type of eugenics provides for genetic manipulation, either in embryos before their transference to a womb or in the gametes used for reproduction. This has been characterized as more radical because the genetic modification is passed on to future generations, and thus any eventual injury derived from the (still) unpredictable effects of this technique are also transmitted (Lanphier *et al*., 2015). In a further association with science fiction, the new technique CRISPR-Cas9, operating by means of a gene-snipping enzyme, can target specific places in an individual’s DNA, rendering this technique a more reliable method of gene editing (Zang & Chen, 2021).

Private eugenics faces several limitations imposed by a lack of scientific knowledge. There are many features that science still cannot shape, particularly personality traits such as kindness or a sense of humor (Turkheimer, 2019). Today scientists can determine sex, eye and hair color, but in many jurisdictions, namely in Europe, laws forbid this type of selection due to ethical, legal and scientific limitations.

## PRIVATE EUGENICS AND SAFETY CONCERNS

Scientific limitations refer to the things that we cannot yet do and to the ones that we can; but not in a safe way. Safety concerns vary according to the specific procedure used, because some of them present serious threats to safety, while others do not create added risks. This is the case of the use of donated (that is, selected) gametes, a procedure that is actually safer than the use of our own gametes, because we rarely test our genetic material. However, in the case of donated gametes significant genetic tests are performed, thus enabling the rejection of gametes not suited for reproduction (Payne *et al*., 2021).

Today, PGD is commonly accepted as a safe practice. There have been concerns about the consequences of removing cells from a developing embryo (Sanders & Griffin, 2017), namely failures in implantation, miscarriages and neurodegenerative disorders (Chen *et al.,* 2018). However, other experts state that blastocyst stage biopsy has a minor impact on an embryo’s viability (Cimadomo *et al*., 2016), and more recent studies even suggest alternative methods to performed preimplantation genetic tests without using biopsies (Aizer *et al*., 2021). It is still unknown whether long-term neurodevelopmental outcomes are affected by the removal of cells at such an early stage (Schendelaar *et al*., 2013). The fact is that safety issues do not seem to affect the use of PGD as much as other concerns of a more legal and ethical nature, such as the destruction of the discarded embryos, or the selection of a specific type of child.

Gene editing is, by far, the most challenging procedure in terms of safety. This conclusion is valid even for the most promising method of gene editing, CRISPR-Cas9 - still under development (Committee on Science, Technology, and Law *et al*., 2016; Yip, 2020). The major risks are the so-called ‘off-target mutations’, that is, unexpected and undesired changes to a gene, which consequences are still unknown (Garrood *et al*., 2021). However, even when the genetic intervention reaches the desired target, there is the risk of unpredictable consequences derived from the modification of the genetic code. In somatic genetic interventions, any eventual detrimental effects only affect the person subjected to them, but in germinal interventions any genetic mistake may have long lasting and serious effects in an undefined number of future generations.

In sum, safety concerns are still a powerful obstacle against the use of private eugenics through gene editing and eventually PGD. Therefore, the use of these procedures in human beings is currently limited, at least in Europe, where gene editing is only allowed in somatic interventions for therapeutic purposes; PGD can only be used when the objective is to prevent the birth of a child with a serious medical condition, because only in that scenario is the risk worth taking (some jurisdictions also allow the use of PGD to raise a child to be a donor of genetic material for an existing person).

I believe that in the near future safety issues will no longer pose a problem, and the only remaining obstacles will be that of an ethical and legal nature. Safety was also a pressing concern when some procedures, which are now a part of current medical practice, started to be used, as in the case of artificial insemination and *in vitro* fertilization; therefore, it is likely that both PGD and gene editing will reach safety levels similar to those other techniques (actually, PGD is already a regular procedure for identifying the presence of certain medical conditions, in spite of the already mentioned safety issues).

However, even if it is considered safe that does not mean that these procedures can be used for every single purpose without further discussion, as demonstrated by the current legal solution regarding gamete donation: even though this does not raise safety problems, its use is generally restricted to cases in which prospective parents are unable to procreate or those in which the resulting child is expected to carry a serious disease (however, in more liberal jurisdictions - even in Europe - gamete donation is allowed in some jurisdictions for single women and same jurisdictions for single women and same sex female couples) these are still frequent restrictions worldwide). In sum, safety is only one minor part of the discussion (and probably the one that will be solved first), so this paper analyzes the other concerns raised by private eugenics.

## THE FUTURE: THE USE OF TORT LAW IN PRIVATE EUGENICS

If parents can select their offspring’s characteristics, we must be prepared for lawsuits presented by parents whose children are born without the previously selected features (Raposo, 2021b). Suppose that someone picks a donor with red hair and freckles because he or she desires those traits in the baby, but the child is born with dark hair and skin. Alternatively, what if someone picks a donor with a very high IQ, but the child presents a very low one?

Lawsuits of this kind are not unprecedented. This type of litigation has frequently occurred when a child is born with a medical condition, despite the precautions taken to prevent it - such as screening supposedly healthy donors that have nonetheless transmitted a disorder to the child (wrongful birth actions). Assuming those events continue to progress in this way, we can get to a point where the choice or determination of the best possible characteristics for an offspring becomes a parental duty (Savulescu, 2001). This is not that different from family law, where violations may result in the lawful removal of parental power.

A case on point is *Harnicher v. University of Utah Medical Center* 962 P.2d 67 (Utah, 1998) (Snow, 2000), in which a couple sought a male donor (donor 183) resembling the male in the couple, who was infertile. However, the resulting children (triplets) did not present any physical similarity to their father or the donor because the fertility clinic (as later discovered) changed the donor from 183 to 83. Instead of curly dark hair and brown eyes, like the father and donor 183 had, the children had straight auburn hair and green eyes, like donor 83, and one of the triplets even had red hair. The plaintiffs’ claim for compensation was denied. The Utah Supreme Court concluded that

there has been no physical harm or injury sustained by the plaintiffs that would enable them to maintain an action for negligent infliction of emotional distress

and the plaintiffs’ alleged physical symptoms were transitory, temporary, and not the kind of physical manifestations of a mental illness that provide the basis for a claim of negligent infliction of emotional distress.

However, it should be noted that in this case the parents’ pretension was not to have a child with certain features just because they were the preferred ones. Rather, their claim was based on the desire for offspring as close as possible to the parents’ physiognomy. Thus, their goal was slightly different than the one discussed in this paper. The harm that can be identified in such a situation is ‘the wrongful denial of legitimate expectations of control over reproductive autonomy and its consequences for parental lives’ (Fox, 2016). This is not an action for wrongful birth but rather a specific kind of wrongful fertilization, in which the injury is not the lack of an expected genetic connection (given that the parents knew from the beginning that they were resorting to a donor). In other words, the injury emerges from wrongful donor selection and consequently from wrongful offspring selection.

## FINAL REMARKS

Eugenics is a natural aspiration, having been with mankind since the dawn of time. The new eugenics is not performed by the State but by private actors. There has been a transition from public interest in improving humankind to parents’ private interest in having a healthy baby or a baby with certain characteristics, aimed at satisfying private desires and the well-being of private individuals. Therefore, in the past, eugenics concerned the sacrifice of reproductive rights, while today it seems to act as an impellent of those rights and a mechanism of reproductive freedom. The challenge is to understand whether we are still talking about reproductive rights or whether these parental choices may represent an abuse of rights.
